# Neuroinflammation modifies the relationship between stress and perivascular spaces in an elderly population with different levels of cognitive impairment

**DOI:** 10.3389/fncel.2024.1480405

**Published:** 2024-11-13

**Authors:** Francesca Sibilia, Nasim Sheikh-Bahaei, Wendy J. Mack, Giuseppe Barisano, Jeiran Choupan

**Affiliations:** ^1^Laboratory of Neuro Imaging, USC Mark and Mary Stevens Neuroimaging and Informatics Institute, Keck School of Medicine, University of Southern California, Los Angeles, CA, United States; ^2^Department of Neurology, Keck School of Medicine, University of Southern California, Los Angeles, CA, United States; ^3^Department of Radiology, Keck School of Medicine, University of Southern California, Los Angeles, CA, United States; ^4^Department of Population and Public Health Sciences, Keck School of Medicine, University of Southern California, Los Angeles, CA, United States; ^5^Department of Neurosurgery, Stanford University, Stanford, CA, United States; ^6^NeuroScope Inc,Scarsdale, NY, United States

**Keywords:** perivascular spaces, neuroinflammation, cognitive impairment, stress, blood–brain barrier, Alzheimer’s disease, glymphatic system, biomarkers

## Abstract

**Background:**

Perivascular spaces (PVS) are fluid-filled spaces surrounding the brain parenchymal vasculature. Literature suggests that PVS may play a significant role in aging and neurological disorders, including Alzheimer’s disease (AD). The aim of this study is to investigate whether the relationship between MRI-visible PVS and stress is influenced by neuroinflammation in an elderly population with different levels of cognitive impairment.

**Methods:**

Using brain MRI scans acquired at 1.5 T, PVS were quantified in a cohort of 461 individuals, consisting of cognitively healthy controls (*n* = 48), people with mild cognitive impairment (MCI, *n* = 322) and Alzheimer’s disease (AD, *n* = 91). PVS volume fraction was calculated in the basal ganglia and centrum semiovale using a semi-automated segmentation approach. Stress was quantified with levels of salivary cortisol. Inflammatory biomarkers measured from plasma included cytokines, matrix metalloproteinases and C-reactive protein. General linear models were used to test the relationship between PVS and cortisol, when interacting with inflammatory markers. This was done on the whole cohort and within each clinical cognitive group.

**Results:**

In the centrum semiovale, higher inflammation levels reduced the relationship of cortisol with PVS. In basal ganglia, higher levels of C-reactive protein reduced the negative relationship of cortisol with PVS. All analyses were accounted for age, sex, body mass index (BMI) and total hippocampal volume. There was a significant interaction effect between cortisol and C-reactive protein on PVS volume fraction in the MCI group.

**Discussion:**

These findings suggest an influence of neuroinflammation on the PVS structure in Alzheimer’s disease spectrum, and offer insight for better understanding physiological processes of cognitive impairment onset.

## 1 Introduction

**Figure 1:**
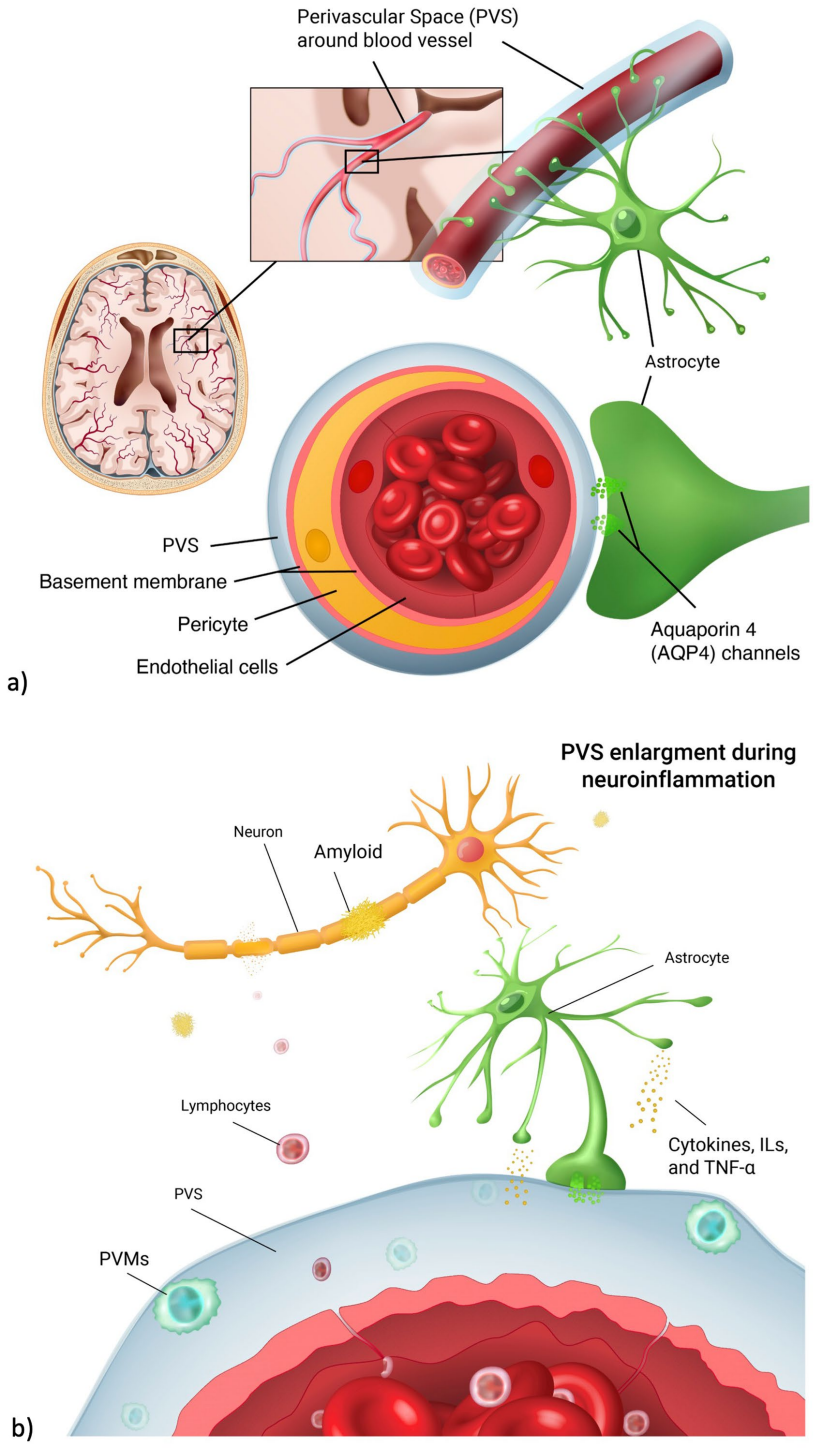
Perivascular space and neuroinflammation. (a) Anatomical representation of the perivascular space (PVS) in the brain vasculature. PVS surrounds the blood vessel lumen. It is involved in the fluid exchange between blood vessels and brain parenchyma through the aquaporin 4 channels, located on the astrocyte endfeet. (b) During neuroinflammation, lymphocytes cross the endothelial cell layer as a result of blood brain barrier leakage. Perivascular macrophages (PVMs) then form, leading to PVS enlargements. As a result, there is a higher accumulation of neurotoxins such as amyloid beta, leading to neuronal death.

Perivascular spaces (PVS) are fluid-filled spaces in the brain surrounding blood vasculature (Doubal et al., 2010). Their function is thought to facilitate the drainage of cerebro-spinal fluid (CSF) between the interstitial space and the blood vessel space, allowing the outflow of waste-soluble proteins from the brain (Zong et al., 2020). PVS are a key part of the glymphatic system, hypothetically responsible for cleansing the brain of neurotoxins (Figure 1a). In healthy populations, an increase in PVS number and volume is seen on brain MRI during normal aging (Lynch et al., 2022), especially in basal ganglia, centrum semiovale and hippocampus (Wardlaw et al., 2020). In clinical populations, studies have shown an association between dilated PVS and severity of neurological diseases, including Huntington’s disease (Chan et al., 2021), cerebral small vessel disease (Brown et al., 2018), and Alzheimer’s disease (AD) (Boespflug et al., 2018).

Increased PVS volume and number might be caused by the alteration of CSF flow in the interstitial space (Barisano et al., 2022). Among environmental and external factors disrupting this balance, psychosocial stress has been found to delay the hemodynamic response and neurovascular coupling that regulate cerebral flow of metabolites needed for cerebral function (Dunlop and Liston, 2018). Higher levels of stress were also linked to hyperproduction of tau and amyloid, neuropathological changes typical of dementia and cognitive impairment (Ennis et al., 2017). Chronic and acute stress have distinct effects on inflammation and brain health. Acute stress activates the hypothalamic–pituitary–adrenal (HPA) axis (Justice, 2018), leading to a temporary increase in cortisol and release of acute inflammatory markers like C-reactive protein (CRP), which helps regulate immune responses. In contrast, chronic stress results in prolonged cortisol exposure, that can disrupt immune function and compromise the integrity of the blood–brain barrier (BBB) promoting neuroinflammation. Increased permeability of the BBB allows immune cells and inflammatory mediators to enter the brain, contributing to structural changes, such as PVS enlargement. This enlargement is associated with impaired drainage of interstitial fluid and accelerated accumulation of waste products (Figure 1b), further exacerbating neuroinflammatory processes and potentially accelerating neurodegenerative diseases.

High cortisol levels can affect blood pressure (BP) and cause hypertension (Ouanes and Popp, 2019). High BP induces a change in vessel dynamics that reduces perivascular pumping, and decreases the net flow of CSF in PVS, with a consequent reduction of parenchymal waste transport (Ennis et al., 2017). Blood pressure is regulated by the renin-angiotensin-adrenal system (RAS). Angiotensin-converting enzyme (ACE) is an enzyme that is part of RAS and is involved in the production of Angiotensin II; high levels of Angiotensin II cause arterial stiffness and structural remodeling, which lead to hypertension and enlarged PVS (Mestre et al., 2017). Increased ACE activity was found to be correlated with parenchymal A β load in AD (Mestre et al., 2017).

Together with the release of cortisol, the interaction of pro-inflammatory factors with the RAS is also crucial for the maintenance of brain vasculature (Xue et al., 2020). ACE has been shown to exert a damaging proinflammatory action on the endothelial and vascular smooth muscle cells (Dandona et al., 2007) that contributes to the onset of cerebral small vessel diseases by disrupting blood brain barrier. Matrix metalloproteinases (in particular MMP-2 and MMP-9) were found to be related to hypertension (Cancemi et al., 2020) and overexpressed around astrocytes in Alzheimer’s disease, promoting accumulation of amyloid beta (A β) plaques. MMP-2 and MMP-9 expression is regulated by tumor necrosis factor alpha (TNF- a ); in turn, the action of TNF- a , TNF-receptor-2 (TNFr2) and TNF-receptor-apoptosis-inducing-ligand (TRAIL), interleukin-6 receptor (IL-6r) and C-reactive protein (CRP) plays a role in the immune system response to physiological and pathological alterations (Burgaletto et al., 2020).

A perturbation in the physiological level of biomarkers in the brain can lead to alterations of the clearance system, where PVS is a crucial component. Exploring the relationship between alterations in PVS volume and plasma biomarkers of inflammation may help understand the mechanisms of neuroinflammation that contribute to the onset of cognitive impairment and neurodegenerative diseases; these mechanisms are understudied so far.

The objective of this study is to investigate the associations of plasma biomarkers of stress and inflammation with PVS volume fraction in older participants of the Alzheimer’s Disease Neuroimaging Initiative 1 (ADNI-1) cohort. Specifically, we tested whether PVS associations with cortisol are influenced by levels of inflammatory biomarkers (TNF- a , TNFr2, TRAIL, CRP, IL-6, MMP-2 and MMP-9). A description of each biomarker is reported in Table 1.

**Table 1:** Description of the stress-related, hypertension and inflammatory markers considered in this study.

Biomarker	Abbreviation	Description
Cortisol	Cort	Final production of the hypothalamic–pituitary–adrenal (HPA) axis, it is defined as the primary stress hormone
Angiotensin converting enzyme	ACE	It is part of the renin-angiotensin-aldosterone-system (RAAS), which regulates blood pressure and vasoconstriction. It converts Angiotensin I in Angiotensin II
Metalloproteinase 2	MMP-2	Enzyme involved in the regulation of vascularization and inflammatory response
Metalloproteinase 9	MMP-9	Enzyme involved in activation of certain interleukins, cleavage of chemokines, and angiogenesis
Interleukin-6 receptor	IL-6r	Cytokine that is involved in cell growth, apoptosis and immune system. It is expressed on brain capillary endothelial cells
C-reactive protein	CRP	Protein produced by the liver. It indicates acute state of inflammation, or used to monitor chronic inflammatory diseases
Tumor necrosis factor alpha	TNF-α	Produced by macrophages during acute inflammation; involved in immunostimulation, resistance to infections
Tumor necrosis factor receptor like 2	TNFr2	Membrane receptors that bind TNF-α
TNF-related apoptosis-inducing ligand	TRAIL	Cytokine released by the majority of tissue cells; they induce cell apoptosis, for example in tumor cells

The table includes the names of the biomarkers in the first column, their corresponding abbreviations in the second column, and a delineation of their physiological functions in the third column.

To our knowledge, this is the first study evaluating associations of stress-related and inflammatory plasma biomarkers with PVS volume fraction in an elderly population, using a novel semi-automated segmentation technique to measure PVS.

## 2 Materials and methods

### 2.1 Participants

Data used in the preparation of this article were obtained from the Alzheimer’s Disease Neuroimaging Initiative (ADNI) database (adni.loni.usc.edu). The ADNI was launched in 2003 as a public-private partnership, led by Principal Investigator Michael W. Weiner, MD. The primary goal of ADNI has been to test whether serial magnetic resonance imaging (MRI), positron emission tomography (PET), other biological markers, and clinical and neuropsychological assessment can be combined to measure the progression of mild cognitive impairment (MCI) and early Alzheimer’s disease (AD).

Participants were older adults from the ADNI-1 population (*n* = 461, age range = 55–90). Inclusion and exclusion criteria can be found on the ADNI dataset manual.^1^ In brief, ADNI inclusion criteria included age between 55 and 90, not enrolled in other studies, generally healthy, fluency in English/Spanish and Geriatric Depression Scale less than 6. Exclusion criteria included the use of specific medications within 4 weeks of screening, such as antidepressants, narcotic analgesics, and anti-Parkinsonian medications with anti-cholinergic activity. Patients with stroke or CAA were also excluded.

The selection of participants for this analysis included those who had complete demographic and plasma biomarker information at the 12-month assessment from the baseline diagnosis. Participants were excluded if they were missing data on age, sex, medication history, biological biomarkers or missing T1w-MPRAGE scans at month-12. From an initial number of 697 that had MRI scans acquired at 1.5 T, no cases were missing for demographics; there were 232 cases with missing biomarker information; for 4 people the PVS segmentation pipeline was not successful; therefore, our final sample size was *n* = 461. The majority of the scans of participants in ADNI 1 were acquired at 1.5 T; plasma biomarkers used in this study (i.e., cortisol, ACE and inflammatory markers) were collected in ADNI 1.

Demographic, biomarker and imaging data were downloaded from the ADNI website.^2^ Participants were categorized based on their diagnosis at ADNI enrollment into cognitively intact healthy controls (CN), persons with mild cognitive impairment (MCI) and persons with more advanced Alzheimer’s diseases (AD). Body mass index (BMI) was considered a hypertension-related factor, expressed as weight (kg) divided by height (m^2^). The number of APOE-4 allele copies was included as a marker of genetic predisposition for AD. Medications were dummy coded based on the type of medication participants reported using (ACE-inhibitors, steroids, other hypertension-related medication). Detailed demographic information of the whole cohort and divided by clinical diagnosis are shown in Table 2.

**Table 2:** Demographic information about ADNI-1 participants.

Variable	ADNI-1, *N* = 461	CN, *n* = 48	MCI, *n* = 322	AD, *n* = 91
Sex				
Male	284 (62%)	26 (54.1%)	209 (64.3%)	52 (56.5%)
Female	177 (38%)	22 (45.8%)	116 (35.7%)	40 (43.5%)
Age (mean ± SD)	75 ± 7	75 ± 5	75 ± 7	75 ± 8
Body mass index (BMI, kg/m^2^)				
<25	194 (41.7%)	15 (31.2%)	140 (43.7%)	39 (43.5%)
25 < 29.9	195 (42.8%)	24 (50%)	132 (40.6%)	39 (42.4%)
≥30	72 (15.5%)	9 (18.8%)	49 (15.07%)	13 (14.1%)
Missing	1		1	
Diagnosis				
AD	91 (20%)			
MCI	322 (70%)			
CN	48 (10%)			
Medication history (ever used)				
(No medication)	404 (87.6%)	40 (83.3%)	285 (88.5%)	79 (86.8%)
(ACE-inhibitors)	30 (6.5%)	4 (8.3%)	20 (6.21%)	6 (6.59%)
(Steroids)	19 (4.12%)	5 (10.42%)	11 (3.42%)	3 (3.3%)
(Other hypertension meds)	16 (3.47%)	2 (4.17%)	9 (2.79%)	5 (5.49%)
APOE4 (copies of alleles)				
0 copy	219 (48%)	43 (89.6%)	147 (46.15%)	29 (31.87%)
1 copy	180 (39%)	5 (10.4%)	133 (40.9%)	42 (46.15%)
2 copies	62 (13%)	0	84 (25.85%)	20 (21.98%)
Systolic blood pressure (mm Hg)				
<120	87 (19.1%)	10 (21%)	63 (19%)	14 (15%)
120–139	200 (43%)	22 (46%)	142 (44%)	35 (38%)
140–159	142 (30.9%)	12 (25%)	94 (29%)	37 (41%)
≥160	30 (0.06%)	4 (8%)	21 (6%)	5 (6%)
Missing	2		2	
Diastolic blood pressure (mm Hg)				
<80	319 (69%)	31 (77%)	223 (69%)	65 (71%)
80–89	122 (26.6%)	17 (23%)	85 (26%)	20 (22%)
90–99	18 (0.039%)	0	12 (4%)	6 (7%)
≥100	0	0	0	0
Missing	2		2	

Five people took more than one type of medication.

### 2.2 Plasma inflammatory and physiological biomarkers

The plasma biomarkers used in this study (cortisol, ACE, TNF- a , TRAIL, TNFr2, CRP, MMP-2 and MMP-9) were collected as part of the Biomarkers Consortium Plasma Proteomics Project RBM multiplex data.^3^ A 190-analyte multiplex immunoassay panel was developed on the Luminex xMAP platform by Rules-Based Medicine (RBM, Austin, TX). The panel, referred to as the human discovery map, contains plasma proteins previously reported in the literature to be altered as a result of cancer, cardiovascular disease, metabolic disorders or inflammation. The Luminex xMAP technology uses a flow-based laser apparatus to detect fluorescent polystyrene microspheres which are loaded with different ratios of two spectrally distinct fluorochromes. Using a ratio of the fluorochromes, up to 100 different beads can be generated such that each of them contains a unique color-coded signature. The beads are bonded with either ligand or antibodies and then standard sandwich assay formats are used to detect the analytes. The beads are read one at a time as they pass through a flow cell on the Luminex laser instrument using a dual laser system. One laser detects the color code and the other reports biomarker concentration. Each plasma biomarker was quantified from blood collected in the morning and is expressed in ng/ml units. The 12-month visit biomarkers measures were correlated with imaging data collected at the same visit.

### 2.3 Medication use

Since both antihypertensive medications and steroids can influence blood pressure and cortisol levels, the history of hypertension-related medication use was evaluated for each participant. ACE-inhibitors were of particular interest as hypertension-related drugs, as their mechanism of action can influence physiological ACE levels. In ADNI-1, each participant provided information on the type of medications ever taken, the dose, the clinical condition for which it was prescribed, and whether they were currently taking the medication at the time of the study visit. Each hypertension medication was assigned to a specific category (ACE-inhibitors, steroid or other hypertension-related medications). A medication variable defined whether each participant ever used a medication related to hypertension or cortisol levels, in particular ACE-inhibitors, steroids (that control cortisol levels), or other hypertension-related medication (0 = ‘no medication’; 1 = ‘ACE-inhibitors’, 2 = ‘steroids’, 3 = ‘other hypertension medications’). Secondly, a 3-level variable was applied to indicate whether participants were currently using any of these medications (0 = ‘no medication history’, 1 = ‘past use of medications’; 2 = ‘current use of medications’).

### 2.4 Imaging

#### 2.4.1 Data acquisition

T1w MPRAGE images (*n* = 465) were acquired using scanners from GE Healthcare, Philips Medical Systems, or Siemens Medical Solutions at 1.5 T (voxel resolution was 1.25×1.25×1.2 mm^3^), TR = 2,400–2,500 ms, TE = 3.6 ms, flip angle = 8–9 degree, FOV = 24–25 cm, slice thickness = 1.2 mm.

#### 2.4.2 Data processing

PVS segmentation followed a previously published technique from our lab (Sepehrband et al., 2019, 2021). In brief, after data wrangling and preprocessing, PVSs were mapped from T1w images: a non-local mean filtering method was applied to denoise the MRI T1w images (Manjón et al., 2010) which is based on how a voxel of interest is similar to neighboring voxels based on their intensity values. A filtering patch with a radius of 1 voxel was applied to retain PVS voxels while removing the image noise at a single-voxel level (Manjón et al., 2010). The Rician noise of MRI scans was considered as the noise reference level for the filtering algorithm. After this, a Frangi filter was applied (Frangi et al., 1998) to detect tubular structures (in this case PVS) on the T1w at a voxel level using the Quantitative Imaging Toolkit (QIT) (Cabeen et al., 2018). A range of 0.1–5 voxels was chosen for this step, as it enhances the detection of vessel-like structures. The output of this step is a probabilistic map of vesselness (Frangi et al., 1998), which was thresholded to obtain a binary PVS mask. The threshold of 0.00008 for the white matter and 0.00015 for the basal ganglia, respectively, was used based on expert opinion to capture true positives and true negatives. White matter lesions (WML) were also removed to eliminate false positives in the PVS segmentation. WML were segmented using a previously validated approach on T1-weighted images: this contrast-adaptive method is based on a generative approach, in which a forward probabilistic model is inverted to obtain automated and robust segmentations of WML (Cerri et al., 2021).

Freesurfer (v.7.1.1) was run to perform image pre-processing (motion correction and image normalization and skull stripping) and to obtain brain volume and parcellation, by running the *recon-all* module on the Laboratory of Neuro Imaging (LONI) pipeline system.^4^ PVS volumes were extracted from both total white matter and basal ganglia (BG), as well as for brain regions based on the Freesurfer’s Desikan-Killiany-Tourville adult cortical parcellation atlas (Klein and Tourville, 2012). The centrum semiovale (CSO) was selected because this region has been historically used as the clinical ROI to assess PVS; PVS volume in the CSO was obtained by adding regions parcellated by the Desikan-Killiany atlas. The regions forming the CSO are listed in Table A1 and Figure A1 (the red-green-blue (RGB) values indicated in the table are from the Freesurfer atlas).

For CSO and basal ganglia, the PVS volume fraction was calculated by dividing the PVS volume by the total volume of the region in each participant (PVS distribution is reported in Figure A2 for both regions). Total hippocampus volume was obtained by Freesurfer volumetric analysis and was used as an indicator of AD-related brain atrophy. A visual example of the PVS segmentation is shown in Figure A3.

### 2.5 Statistical analysis

PVS volume fractions for both basal ganglia and CSO were used as a dependent variable (DV). The primary independent variable (IV) of interest for each model was represented by the interaction between cortisol (as a measure of stress) and inflammatory biomarkers. To model the positive, continuous skewed PVS data, we used generalized linear regression specifying a Gamma distribution and log link function. Distributions of the inflammatory biomarkers are reported in Figure A4. Continuous independent variables used in interaction analyses (cortisol and inflammatory markers) were centered around their median values. Sex, age, plasma ACE, BMI, and hippocampal volume were included as covariates in all analyses. In sensitivity analyses, we also included an indicator variable for APOE4 positivity as a model covariate. Models were re-run including medications also as covariates, coding them as factor variables as described in Section 2.3, to investigate whether including medications as a covariate would change the results of our models. The interaction terms in the Gamma regression models are described by the mathematical formula below. The statistical models were run considering separately the PVS volume fraction ( $\mathrm{pv}s_{vf})$ of the centrum semiovale and basal ganglia as dependent variables. Equation 1 depicts our primary analysis regression model. The log of the mean PVS volume fraction (dependent variable) is modeled as a function of (1) primary independent variables of interest (inflammation, cortisol); (2) adjusting covariates (age, sex, ACE, BMI, hippocampal volume); (3) a product interaction term of cortisol*inflammation. Regression coefficients test the association of each independent variable with log(mean PVS volume fraction); beta estimates that are not significantly different from zero indicate no association with PVS volume fraction. In the presence of the cortisol-inflammation interaction term, the main inflammation term tests the association of inflammation with log(mean PVS volume fraction) in persons at the median level of cortisol (i.e., median-centered cortisol = 0). The regression coefficient for the product interaction term tests if the association of inflammation with PVS volume fraction changes with level of cortisol; for example, a positive regression coefficient indicates that the association of inflammation with log(mean PVS volume fraction) is more strongly positive as cortisol levels increase:


(1) $$\begin{array}{l} \log\;\left(E\left[\mathrm{pv}s_{vf}\right]\right)=b_{0}+b_{1}\left(\mathrm{median}-\mathrm{centered}\;\mathrm{Cortisol}\right)\\ \;+b_{2}\left(\mathrm{median}-\mathrm{centered}\;\mathrm{Inflammatio}n_{\mathrm{biomarker}}\right)\\ \;+b_{3}(\mathrm{median}-\mathrm{centered}\;\mathrm{Cortisol}*\mathrm{median}\\ \;-\mathrm{centered}\;\mathrm{Inflammatio}n_{\mathrm{biomarker}})\\ \;+b_{4}\left(\mathrm{ACE}\right)+b_{5}\left(Sex_{M/F}\right)+b_{6}\left(\mathrm{Age}\right)\\ \;+b7\left(BMI\right)+b8\left(\mathrm{Hippocampu}s_{\mathrm{vol}}\right)+\varepsilon \text{.} \end{array}$$

**Equation 1:** Regression models describing the interaction between plasma cortisol and inflammatory markers; they were run for both PVS volume fraction in centrum semiovale and basal ganglia.

The same models were run within each cognitive diagnosis group (AD, MCI, CN).

For statistical analysis, a *glm* function with Gamma family and log link was run in RStudio (v.2022.07.2). To illustrate interactions, we showed plots of statistically significant fitted associations of cortisol at two levels (minimum and maximum) of inflammatory markers. We utilized the Benjamini–Hochberg (BH) false discovery rate (FDR) approach (Benjamini and Hochberg, 1995) to adjust for multiple hypothesis testing of cortisol interactions with inflammatory markers, with alpha value of 0.05; FDR testing was completed separately within each dependent variable.

### 2.6 Standard protocol approvals, registrations, and patient consents

Approval to use ADNI data was obtained by sending a request online to ADNI. The ADNI study was approved by the Institutional Review Boards of all the participating institutions. Informed written consent was obtained from all participants at each site. More details can be found at https://adni.loni.usc.edu/.

## 3 Results

Demographic information for both the whole sample and divided by clinical diagnosis is reported in Table 2. There was no significant association between PVS volume fraction and cortisol levels (in both CSO and BG), either in the whole sample (results are reported in Table A2) or within cognitive diagnosis groups (*p* > 0.2 adjusted for ACE, age, sex, BMI, hippocampal volume).

### 3.1 Cortisol-inflammation interactions

#### 3.1.1 Centrum semiovale (CSO-PVS) and cortisol

**Figure 2:**
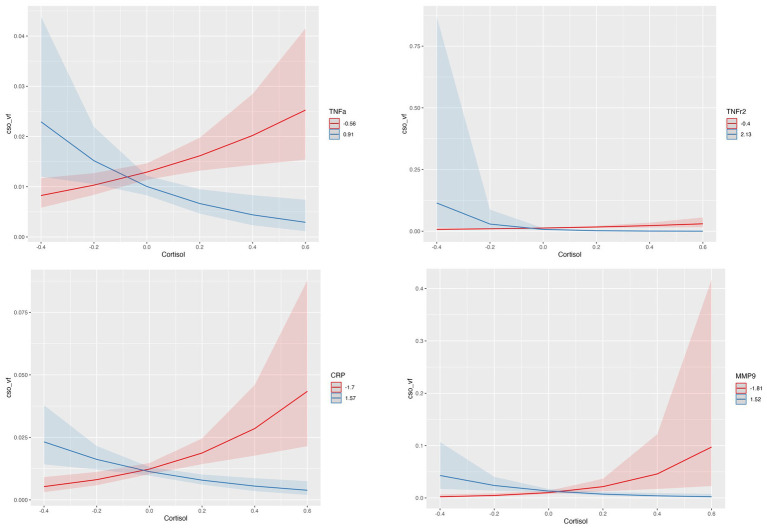
Statistically significant interactions between Cortisol and TNFr2, TNF- α , CRP and MMP-9 in centrum semiovale (CSO) PVS volume fraction (vf). Plots indicate model-fitted cortisol-PVS curves at two different levels of each inflammatory biomarker. The two levels of inflammatory markers indicate the minimum and the maximum values. Shaded areas represent 95% confidence intervals.

Interactions of cortisol with inflammatory biomarkers showed inverse associations with CSO-PVS, indicating that higher levels of inflammation reduced positive associations of cortisol with PVS. At lower levels of neuroinflammation, higher levels of cortisol are associated with enlargement of PVS volume fraction; when neuroinflammatory biomarkers levels are higher, higher levels of cortisol are associated with decreases in PVS volume fraction. This interaction effect was observed for TNFr2 (*q* value = 0.0106), TNF- α (interaction *q* value =0.011), CRP (*q* value = 0.0036) and MMP9 (*q* value = 0.0106) (Figure 2). The model used was:


$$\begin{array}{l} \log\;\left(E\left[CSO\_\mathrm{pv}s_{vf}\right]\right)=b_{0}+b_{1}(\mathrm{median}-\mathrm{centered}\;\mathrm{Cortisol}\\ \;*\mathrm{median}-\mathrm{centered}\;\mathrm{Inflammatio}n_{\mathrm{biomarker}})\\ \;+b_{2}\left(\mathrm{ACE}\right)+b_{3}\left(Sex_{M/F}\right)+b_{4}\left(\mathrm{Age}\right)\\ \;+b5\left(BMI\right)+b6\left(\mathrm{Hippocampu}s_{\mathrm{vol}}\right)+\varepsilon \end{array}$$

Regression model results are shown in Table 3. The interactions between cortisol and TRAIL, MMP2 and IL-6r were not statistically significant.

**Table 3:** Estimated beta coefficients, standard error, and p-values for significant interactions between cortisol and inflammatory biomarkers (TNF- α , TNFr2, CRP and MMP-9) in the centrum semiovale PVS volume fraction.

*Centrum semiovale (CSO-PVS)*																
Predictors	Estimates	SE	*p*	*q*	Estimates	SE	*p*	*q*	Estimates	SE	*p*	*q*	Estimates	SE	*p*	*q*
Intercept	−6.598	0.49	<0.001		−6.570	0.477	<0.001		−6.531	0.474	**<0.001**		−6.473	0.475	**<0.001**	
Cortisol	0.097	0.184	0.599		−0.093	0.194	0.633		0.074	0.183	0.68		0.118	0.185	0.52	
TNFr2	−0.223	0.132	0.091													
ACE	0.033	0.156	0.83		0.039	0.155	0.8		0.0706	0.154	0.64		0.021	0.155	0.89	
Age	0.019	0.003	**<0.001**		0.018	0.0034	**<0.001**		0.017	0.003	**<0.001**		0.017	0.003	**<0.001**	
Sex [Male]	0.0304	0.049	0.538		0.014	0.049	0.77		0.005	0.05	0.91		0.015	0.049	0.75	
BMI	0.0109	0.005	0.065		0.011	0.005	0.056		0.009	0.005	0.115		0.009	0.005	0.11	
Total HC	0.00006	0.00002	**0.0112**		0.0006	0.0002	**0.009**		0.00006	0.00002	**0.009**		0.00006	0.00002	**0.004**	
Cortisol*TNFr2	−3.2738	1.187	**0.0061**	**0.011**												
TNFa					−0.17	0.096	0.07									
Cortisol*TNFa							**0.005**	**0.011**								
CRP									−0.026	0.046	0.573					
Cortisol*CRP									−1.192	0.341	**0.0005**	**0.0036**				
MMP9													0.079	0.082	0.34	
Cortisol*MMP9													−2.014	0.687	**0.0036**	**0.011**

Uncorrected *p*-values and *q*-values (after FDR correction) are reported (in bold). SE, standard error; HC, hippocampal volume.

Results were confirmed when information on whether participants ever used medications was added to the model.

#### 3.1.2 Basal ganglia (BG-PVS) and cortisol

**Figure 3:**
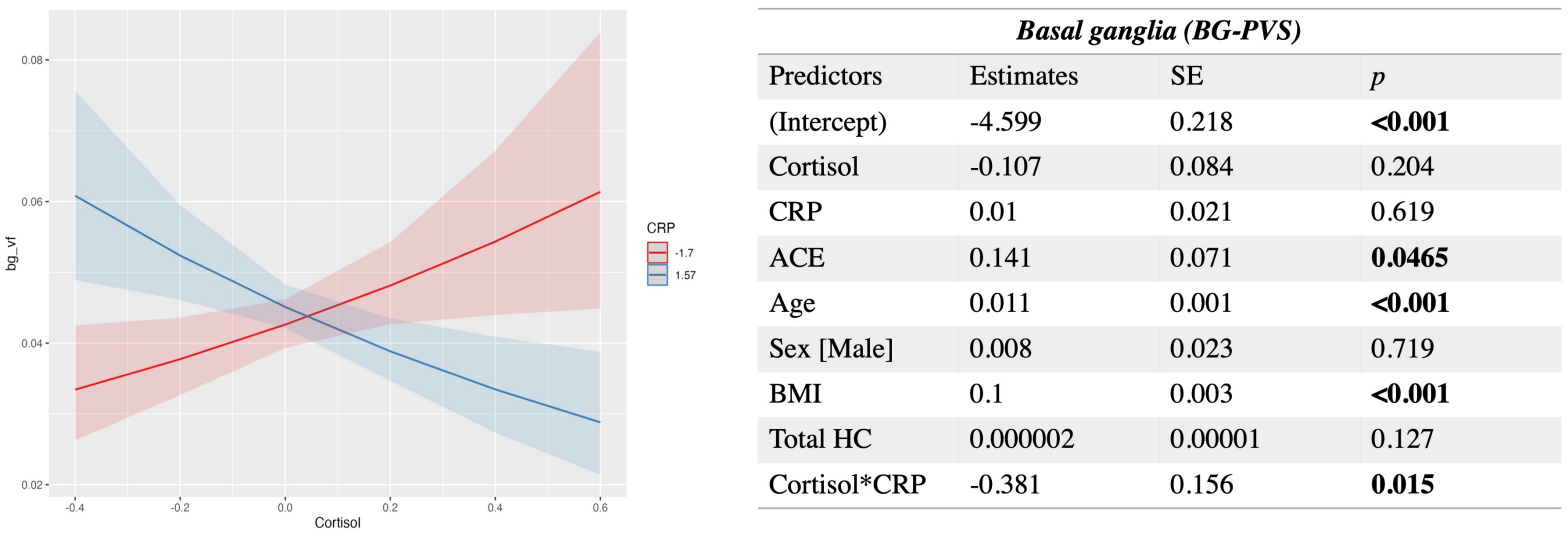
Estimated beta coefficients, standard error, and *p*-values for the significant interaction between Cortisol and CRP in the basal ganglia PVS. SE, standard error; HC, hippocampal volume. Plots indicate model-fitted cortisol-PVS curves at two different levels of each inflammatory biomarker. The two levels of inflammatory markers indicate the minimum and the maximum values. Shaded areas represent 95% confidence intervals.

Results showed significant negative interactions between cortisol and both MMP9 and CRP. After FDR correction, only the interaction between cortisol and CRP remained statistically significant (*q* value = 0.046). At lower levels of CRP, higher cortisol is associated with enlarged BG-PVS volume fraction; when CRP levels increase, higher levels of cortisol are associated with decreased PVS volume fraction. Age, ACE and BMI had significant positive associations with BG-PVS (Figure 3). The model used was:


$$\begin{array}{l} \log\;\left(E\left[BG\_\mathrm{pv}s_{vf}\right]\right)=b_{0}+b_{1}(\mathrm{median}-\mathrm{centered}\;\mathrm{Cortisol}\\ \;*\mathrm{median}-\mathrm{centered}\;\mathrm{Inflammatio}n_{\mathrm{biomarker}})\\ \;+b_{2}\left(\mathrm{ACE}\right)+b_{3}\left(Sex_{M/F}\right)+b_{4}\left(\mathrm{Age}\right)\\ \;+b5\left(BMI\right)+b6\left(\mathrm{Hippocampu}s_{\mathrm{vol}}\right)+\varepsilon \end{array}$$

When we included ApoE4 as a model covariate representing genetic predisposition to Alzheimer’s disease, the presented cortisol-inflammation interaction results did not change either for CSO-PVS or BG-PVS.

### 3.2 Within-cognitive group analysis

To better understand the effect of cognitive impairment on the PVS volume fraction, we conducted within-group analyses by dividing our sample in CN (*n* = 48), MCI (*n* = 322) and AD (*n* = 91) groups. The diagnosis for each participant was based on clinical and syndromic diagnosis, considering subjective memory concerns reported by the participant, study partner or clinician; cut-off scores of cognitive assessments, such as Mini–Mental State Examination (MMSE), Clinical Dementia Rating (CDR), rather than biological markers. In fact, most of the participants in ADNI-1 lacked biological markers information; CSF and PET data were missing for more than 50% of the population we considered. The groups were age and gender equivalent (chi-square test *p*-value = 0.95 and 0.21 respectively). We ran the same regression models as for the entire population.

#### 3.2.1 Healthy controls

Following correction for multiple comparisons, no statistically significant associations with CSO-PVS or BG-PVS were observed within the healthy control group.

#### 3.2.2 MCI

The MCI group represented the majority of our sample (70%). In this group, the level of cortisol was positively associated with higher PVS volume fraction in CSO for cases with lower CRP levels (interaction beta = −1.55, SE = 0.45, *q* value =0.00042); the same was found in the BG (interaction beta = − 0.677, SE = 0.19 *and q* value =0.0043). This indicates that at low levels of CRP, higher levels of cortisol are associated with enlarged CSO-PVS and BG-PVS volume fraction; when CRP levels are higher, cortisol increase is associated with lower PVS volume fraction. Interaction plots are shown in Figure A5.

#### 3.2.3 AD patients

In the AD group, interaction of cortisol with MMP-9 showed inverse associations with CSO-PVS (*p* value = 0.0206, *q* = 0.08), with a positive effect of hippocampal volume (*p* value = 0.015, *q* = 0.08), indicating that at low levels of MMP9, higher levels of cortisol are associated with enlarged CSO-PVS and BG-PVS volume fraction; when MMP9 levels increase, higher levels of cortisol are associated with decreased PVS volume fraction. Such association did not survive correction for multiple comparisons.

## 4 Discussion

We show novel associations between stress, inflammation cytokines, and perivascular space (PVS) volume fraction. The association between neuroinflammation and PVS was recently described by Zeng et al. (2022) as a “vicious cycle.” While the accumulation of inflammatory cellular components can cause PVS enlargements, associated with A β accumulation, neurodegeneration can trigger a neuroinflammatory cascade, linked to further PVS enlargements and decrease of the glymphatic clearance activity.

### 4.1 Interactions with cortisol

In the CSO-PVS, we found statistically significant interactions between cortisol and TNF- α , MMP9, TNFr2 and CRP, suggesting that baseline levels of inflammatory markers modulate the association between cortisol and PVS volume fraction. Older age and hippocampal volume were also showed statistically significant correlates; these variables were previously shown to contribute to PVS enlargement in healthy individuals (Barisano et al., 2021).

In the healthy brain or during short-term stress, cortisol plays a pivotal role as a powerful anti-inflammatory hormone. When levels of inflammatory markers are low, cortisol activity is crucial to ensure an efficient brain clearance, by limiting the spread of damaged cells in the brain, regulating the levels of glucose, and being involved in the suppression of pro-inflammatory cytokines, modulation of immune cell activity, and maintenance of blood–brain barrier (BBB) integrity (Turner et al., 2020; Hannibal and Bishop, 2014).

However, in pathological states, microglia-macrophages can disturb the BBB and neurogenesis by producing excessive cytokines. Chronic inflammation can disrupt the blood–brain barrier and permeability, linked to fluid accumulation and enlargement of the perivascular spaces. During a brain insult, activated macrophages and microglia release pro-inflammatory cytokines and lead to cortisol hyper-production as stress response, contributing to faster A β accumulation and accelerated cognitive impairment (Toledo et al., 2012; Pistollato et al., 2016). In animal studies, the release of cortisol increases the accumulation of tau and beta-amyloid and brain atrophy. In an experimental study, aged mice exhibited elevated proinflammatory cytokines (in particular TNF- a and CRP), HPA axis dysregulation, and reduced glucocorticoid synthesis (Watanabe et al., 2016). The dysregulation of glucocorticoid production (including cortisol) associated with aging has an impact on inflammatory processes and immune system activation (Valbuena Perez et al., 2020). Furthermore, the microglia response to brain insult and neurotoxins accumulation becomes ineffective in chronic stress situations, contributing to cognitive impairment. Once activated, glial cells release cytokines and chemokines into the extra-synaptic space, causing neurotransmitter system dysregulation, an imbalance between excitatory and inhibitory signals, and impairing neural circuitry plasticity and adaptation.

The hippocampus is one of the first regions affected during neurodegenerative processes, with increased oxidative stress. During chronic stress, the hippocampus is also one of the most susceptible brain regions as it contains a high concentration of glucocorticoid receptors (Knezevic et al., 2023). It remains uncertain whether the hyperproduction of glucocorticoids precedes and contributes to hippocampal neuron damage and accelerated atrophy, or if regional atrophy itself leads to dysfunction of the HPA-axis.

Excessive production of cortisol modulates the expression and function of tight junction proteins, associated with increased BBB permeability (Sweeney et al., 2018). This disruption of the BBB can exacerbate neuroinflammation by allowing peripheral immune cells and pro-inflammatory cytokines infiltration. High cortisol levels also impact the perivascular spaces, impairing the brain’s ability to efficiently remove interstitial fluid and metabolic waste, including amyloid-beta (Plog and Nedergaard, 2018). People with cognitive impairments present dysfunctions also in the negative feedback system of the HPA axis (de Souza-Talarico et al., 2011), which leads to higher production of cortisol. Previous studies suggest that cognitive impairment is linked to elevated basal cortisol levels, driven by accelerated cerebral A β accumulation (Pistollato et al., 2016; Boespflug et al., 2018) and enlarged PVS volume fraction (Sepehrband et al., 2021). A previous study showed an association between decreased levels of salivary cortisol and higher levels of neuroinflammatory markers in people with AD (Daniilidou et al., 2023), while MCI showed higher levels of chemokines compared to AD, suggesting a more active role of neuroinflammation at early stages of the disease.

It has been proposed that cortisol plays a dual effect in regulating inflammation processes and immune response (Yeager et al., 2011). Glucocorticoids (GCs), among which cortisol is the most common, have both pro- and anti-inflammatory effects. Optimal glucocorticoid levels are key for effective acute inflammation responses; decreases of GCs trigger prolonged inflammation, while excessive levels impair infection defense and tissue repair. In humans, GCs low levels are associated with a weaker immune response and recurrent infections; this highlights that the balance between glucocorticoids activity and inflammatory mediators’ action is necessary for immune mechanisms and the removal of pathogens (Cruz-Topete and Cidlowski, 2015).

### 4.2 PVS in clinical settings

Many of our results showed statistically significant associations and interactions on the CSO-PVS compared to BG-PVS. While alterations in BG-PVS are more linked to hypertension and small vessel diseases, CSO-PVS structural alterations are associated with cognitive impairment due to A β (Kim et al., 2021). They found a high degree of CSO-PVS was associated to A β positivity in neurodegenerative processes and cognitive impairment. Another study evaluated PVS changes in Asian individuals with intracerebral hemorrhage who developed cerebral amyloid angiopathy (CAA) (Tsai et al., 2021). Their results showed that patients developing CAA had a higher degree of CSO-PVS associated with higher vascular A β retention compared to those with a low-degree CSO-PVS. A high degree of CSO-PVS was also found in microglia-related inflammation in older people (Zeng et al., 2022), suggesting that inflammatory processes can lead to the glymphatic system dysfunction and deposition of Alzheimer’s disease-related biomarkers (i.e., A β and Tau).

We found CRP levels modified cortisol associations with PVS volume fraction, both in basal ganglia and centrum semiovale. CRP is known as a biomarker for acute inflammation but is also involved in chronic inflammation state of neurodegenerative diseases (Noble et al., 2010). Lower levels of CRP have been associated with a cognitive impairment in stroke patients developing dementia after ischemia (Luan and Yao, 2018). In addition, CRP levels differences across individuals were found to be influenced by lifestyle factors, for example obesity (Natale et al., 2022) and ethnicity (Morimoto et al., 2014; Watanabe et al., 2016). Physiologically, CRP levels exhibit stability and lack diurnal variations, indicating its potential as a reliable measure in clinical settings to examine brain vasculature changes associated with neurodegenerative diseases.

In the basal ganglia, advancing age, ACE, and BMI were associated with an increased PVS volume fraction and heightened inflammation. This aligns with previous research indicating a connection between higher BMI and elevated cytokine levels, especially in individuals classified as overweight or obese, presenting excess adipose tissue (Schmidt et al., 2016; Landi et al., 2018). Moreover, mood disorders, including depression, have also been linked to both higher BMI and neuroinflammation (Lorena et al., 2021). It has been proposed that the pro-inflammatory environment in the brain can affect neurotransmitter systems and neural adaptability, potentially contributing to the onset and persistence of mood disorders (Rosenblat et al., 2014). When systemic inflammation occurs, whether due to obesity, hypertension or other factors, these cytokines can breach the blood–brain barrier, trigger the activation of microglia and prolong neuroinflammation.

### 4.3 Strengths and limitations

Most of the literature on neuroinflammation and PVS neuroimaging is based on PVS count using visual rating scores. Our semi-automated technique detects changes in PVS morphology and volume (i.e., enlargement) that reflect waste accumulation in the brain, and therefore represents a methodological strength for this study.

Although the lack of 3D FLAIR images in the ADNI-1 population and the decreased specificity of T1w images with respect to vascular abnormalities can be seen as a limitation, we were able to remove WMLs as false positives. The main results shown in this study considered the population as one group of older individuals, although their diagnoses included CN, MCI and AD. We ran further analyses splitting the population based on diagnosis; the sample size for each group was quite unbalanced (most of our participants were MCIs—70% MCI, 20% AD and 10% CN), and this could affect the results. The lack of significant results in the CN group especially could reflect the small sample size (*n* = 48), rather than having clinical significance. Another limitation is the lack of plasma A β and tau measures in the analysis as markers of Alzheimer’s disease. This was not possible as these data were not available in more than half of participants in the ADNI-1 cohort. We tested for the APOE4 allele presence as a covariate in the model, which did not alter our cortisol-inflammation interaction results.

This study shows how the interaction between inflammation with cortisol is related to PVS volume fraction size in older people with and without cognitive impairment. The concentration of cytokines is crucial for the physiological maintenance of the brain immune response. Future studies should build on these findings to investigate cell populations that are mostly found in PVS during neuroinflammation, and how the accumulation of inflammatory markers can disrupt the fluid exchange weakening the glymphatic system and brain clearance mechanisms. Understanding if higher levels of plasma biomarkers indicating chronic and acute inflammation, such as TNF- a , TNFr2, MMP9 and CRP are related to enlargement of PVS or vice versa can be helpful to develop therapeutic solutions to prevent neurovascular damage.

## Data Availability

Publicly available datasets were analyzed in this study. This data can be found here: https://adni.loni.usc.edu/.

## Footnotes

^1^https://adni.loni.usc.edu/wp-content/uploads/2010/09/ADNI_GeneralProceduresManual.pdf

^2^ http://adni.loni.usc.edu

^3^https://adni.loni.usc.edu/wp-content/uploads/2010/11/BC_Plasma_Proteomics_Data_Primer.pdf

^4^ https://pipeline.loni.usc.edu

## The Alzheimer's Disease Neuroimaging Initiative

Data used in preparation of this article were obtained from the Alzheimer’s Disease Neuroimaging Initiative (ADNI) database (adni.loni.usc.edu). As such, the investigators within the ADNI contributed to the design and implementation of ADNI and/or provided data but did not participate in analysis or writing of this report. A complete listing of ADNI investigators can be found at: http://adni.loni.usc.edu/wp-content/uploads/how_to_apply/ADNI_Acknowledgement_List.pdf. Michael Weiner, MD (UC San Francisco, Principal Investigator, Executive Committee); Paul Aisen, MD (UC San Diego, ADCS PI and Director of Coordinating Center Clinical Core, Executive Committee, Clinical Core Leaders); Ronald Petersen, MD, PhD (Mayo Clinic, Rochester, Executive Committee, Clinical Core Leader); Clifford R. Jack, Jr., MD (Mayo Clinic, Rochester, Executive Committee, MRI Core Leader); William Jagust, MD (UC Berkeley, Executive Committee; PET Core Leader); John Q. Trojanowki, MD, PhD (U Pennsylvania, Executive Committee, Biomarkers Core Leader); Arthur W. Toga, PhD (USC, Executive Committee, Informatics Core Leader); Laurel Beckett, PhD (UC Davis, Executive Committee, Biostatistics Core Leader); Robert C. Green, MD, MPH (Brigham and Women’s Hospital, Harvard Medical School, Executive Committee and Chair of Data and Publication Committee); Andrew J. Saykin, PsyD (Indiana University, Executive Committee, Genetics Core Leader); JohnMorris, MD (Washington University St. Louis, Executive Committee, Neuropathology Core Leader); Leslie M. Shaw (University of Pennsylvania, Executive Committee, Biomarkers Core Leader); Enchi Liu, PhD (Janssen Alzheimer Immunotherapy, ADNI 2 Private Partner Scientific Board Chair); Tom Montine, MD, PhD (University of Washington); Ronald G. Thomas, PhD (UC San Diego); Michael Donohue, PhD (UC San Diego); Sarah Walter, MSc (UC San Diego); Devon Gessert (UC San Diego); Tamie Sather, MS (UC San Diego,); Gus Jiminez, MBS (UC San Diego); Danielle Harvey, PhD (UC Davis;); Michael Donohue, PhD (UC San Diego); Matthew Bernstein, PhD (Mayo Clinic, Rochester); Nick Fox, MD (University of London); Paul Thompson, PhD (USC School of Medicine); Norbert Schuff, PhD (UCSF MRI); Charles DeCArli, MD (UC Davis); Bret Borowski, RT (Mayo Clinic); Jeff Gunter, PhD (Mayo Clinic); Matt Senjem, MS (Mayo Clinic); Prashanthi Vemuri, PhD (Mayo Clinic); David Jones, MD (Mayo Clinic); Kejal Kantarci (Mayo Clinic); Chad Ward (Mayo Clinic); Robert A. Koeppe, PhD (University of Michigan, PET Core Leader); Norm Foster, MD (University of Utah); Eric M. Reiman, MD (Banner Alzheimer’s Institute); Kewei Chen, PhD (Banner Alzheimer’s Institute); Chet Mathis, MD (University of Pittsburgh); Susan Landau, PhD (UC Berkeley); Nigel J. Cairns, PhD, MRCPath (Washington University St. Louis); Erin Householder (Washington University St. Louis); Lisa Taylor Reinwald, BA, HTL (Washington University St. Louis); Virginia Lee, PhD, MBA (UPenn School of Medicine); Magdalena Korecka, PhD (UPenn School of Medicine); Michal Figurski, PhD (UPenn School of Medicine); Karen Crawford (USC); Scott Neu, PhD (USC); Tatiana M. Foroud, PhD (Indiana University); Steven Potkin, MD UC (UC Irvine); Li Shen, PhD (Indiana University); Faber Kelley, MS, CCRC (Indiana University); Sungeun Kim, PhD (Indiana University); Kwangsik Nho, PhD (Indiana University); Zaven Kachaturian, PhD (Khachaturian, Radebaugh & Associates, Inc. and Alzheimer’s Association’s Ronald and Nancy Reagan’s Research Institute); Richard Frank, MD, PhD (General Electric); Peter J. Snyder, PhD (Brown University); Susan Molchan, PhD (National Institute on Aging/National Institutes of Health); Jeffrey Kaye, MD (Oregon Health and Science University); Joseph Quinn, MD (Oregon Health and Science University); Betty Lind, BS (Oregon Health and Science University); Raina Carter, BA (Oregon Health and Science University); Sara Dolen, BS (Oregon Health and Science University); Lon S. Schneider, MD (University of Southern California); Sonia Pawluczyk, MD (University of Southern California); Mauricio Beccera, BS (University of Southern California); Liberty Teodoro, RN (University of Southern California); Bryan M. Spann, DO, PhD (University of Southern California); James Brewer, MD, PhD (University of California San Diego); Helen Vanderswag, RN (University of California San Diego); Adam Fleisher, MD (University of California San Diego); Judith L. Heidebrink, MD, MS (University of Michigan); Joanne L. Lord, LPN, BA, CCRC (University of Michigan); Ronald Petersen, MD, PhD (Mayo Clinic, Rochester); Sara S. Mason, RN (Mayo Clinic, Rochester); Colleen S. Albers, RN (Mayo Clinic, Rochester); David Knopman, MD (Mayo Clinic, Rochester); Kris Johnson, RN (Mayo Clinic, Rochester); Rachelle S. Doody, MD, PhD (Baylor College of Medicine); Javier Villanueva Meyer, MD (Baylor College of Medicine); Munir Chowdhury, MBBS, MS (Baylor College of Medicine); Susan Rountree, MD (Baylor College of Medicine); Mimi Dang, MD (Baylor College of Medicine); Yaakov Stern, PhD (Columbia University Medical Center); Lawrence S. Honig, MD, PhD (Columbia University Medical Center); Karen L. Bell, MD (Columbia University Medical Center); Beau Ances, MD (Washington University, St. Louis); John C. Morris, MD (Washington University, St. Louis); Maria Carroll, RN, MSN (Washington University, St. Louis); Sue Leon, RN, MSN (Washington University, St. Louis); Erin Householder, MS, CCRP (Washington University, St. Louis); Mark A. Mintun, MD (Washington University, St. Louis); Stacy Schneider, APRN, BC, GNP (Washington University, St. Louis); Angela Oliver, RN, BSN, MSG; Daniel Marson, JD, PhD (University of Alabama Birmingham); Randall Griffith, PhD, ABPP (University of Alabama Birmingham); David Clark, MD (University of Alabama Birmingham); David Geldmacher, MD (University of Alabama Birmingham); John Brockington, MD (University of Alabama Birmingham); Erik Roberson, MD (University of Alabama Birmingham); Hillel Grossman, MD (Mount Sinai School of Medicine); Effie Mitsis, PhD (Mount Sinai School of Medicine); Leyla deToledo-Morrell, PhD (Rush University Medical Center); Raj C. Shah, MD (Rush University Medical Center); Ranjan Duara, MD (Wien Center); Daniel Varon, MD (Wien Center); Maria T. Greig, HP (Wien Center); Peggy Roberts, CNA (Wien Center); Marilyn Albert, PhD (Johns Hopkins University); Chiadi Onyike, MD (Johns Hopkins University); Daniel D’Agostino II, BS (Johns Hopkins University); Stephanie Kielb, BS (Johns Hopkins University); James E. Galvin, MD, MPH (New York University); Dana M. Pogorelec (New York University); Brittany Cerbone (New York University); Christina A. Michel (New York University); Henry Rusinek, PhD (New York University); Mony J de Leon, EdD (New York University); Lidia Glodzik, MD, PhD (New York University); Susan De Santi, PhD (New York University); P. Murali Doraiswamy, MD (Duke University Medical Center); Jeffrey R. Petrella, MD (Duke University Medical Center); Terence Z. Wong, MD (Duke University Medical Center); Steven E. Arnold, MD (University of Pennsylvania); Jason H. Karlawish, MD (University of Pennsylvania); David Wolk, MD (University of Pennsylvania); Charles D. Smith, MD (University of Kentucky); Greg Jicha, MD (University of Kentucky); Peter Hardy, PhD (University of Kentucky); Partha Sinha, PhD (University of Kentucky); Elizabeth Oates, MD (University of Kentucky); Gary Conrad, MD (University of Kentucky); Oscar *L. Lopez*, MD (University of Pittsburgh); MaryAnn Oakley, MA (University of Pittsburgh); Donna M. Simpson, CRNP, MPH (University of Pittsburgh); Anton P. Porsteinsson, MD (University of Rochester Medical Center); Bonnie S. Goldstein, MS, NP (University of Rochester Medical Center); Kim Martin, RN (University of Rochester Medical Center); Kelly M. Makino, BS (University of Rochester Medical Center); M. Saleem Ismail, MD (University of Rochester Medical Center); Connie Brand, RN (University of Rochester Medical Center); Ruth A. Mulnard, DNSc, RN, FAAN (University of California, Irvine); Gaby Thai, MD (University of California, Irvine); Catherine Mc Adams Ortiz, MSN, RN, A/GNP (University of California, Irvine); Kyle Womack, MD (University of Texas Southwestern Medical School); Dana Mathews, MD, PhD (University of Texas Southwestern Medical School); Mary Quiceno, MD (University of Texas Southwestern Medical School); Ramon Diaz Arrastia, MD, PhD (University of Texas Southwestern Medical School); Richard King, MD (University of Texas Southwestern Medical School); Myron Weiner, MD (University of Texas Southwestern Medical School); Kristen Martin Cook, MA (University of Texas Southwestern Medical School); Michael DeVous, PhD (University of Texas Southwestern Medical School); Allan I. Levey, MD, PhD (Emory University); James J. Lah, MD, PhD (Emory University); Janet S. Cellar, DNP, PMHCNS BC (Emory University); Jeffrey M. Burns, MD (University of Kansas, Medical Center); Heather S. Anderson, MD (University of Kansas, Medical Center); Russell H. Swerdlow, MD (University of Kansas, Medical Center); Liana Apostolova, MD (University of California, Los Angeles); Kathleen Tingus, PhD (University of California, Los Angeles); Ellen Woo, PhD (University of California, Los Angeles); Daniel H.S. Silverman, MD, PhD (University of California, Los Angeles); Po H. Lu, PsyD (University of California, Los Angeles); George Bartzokis, MD (University of California, Los Angeles); Neill R Graff Radford, MBBCH, FRCP (London) (Mayo Clinic, Jackson ville); Francine Parfitt, MSH, CCRC (Mayo Clinic, Jacksonville); Tracy Kendall, BA, CCRP (Mayo Clinic, Jacksonville); Heather Johnson, MLS, CCRP (Mayo Clinic, Jacksonville); Martin R. Farlow, MD (Indiana University); Ann Marie Hake, MD (Indiana University); Brandy R. Matthews, MD (Indiana University); Scott Herring, RN, CCRC (Indiana University); Cynthia Hunt, BS, CCRP (Indiana University); Christopher H. van Dyck, MD (Yale University School of Medicine); Richard E. Carson, PhD (Yale University School of Medicine); Martha G. MacAvoy, PhD (Yale University School of Medicine); Howard Chertkow, MD (McGill Univ., Montreal Jewish General Hospital); Howard Bergman, MD (McGill Univ., Montreal Jewish General Hospital); Chris Hosein, Med (McGill Univ., Montreal Jewish General Hospital); Sandra Black, MD, FRCPC (Sunnybrook Health Sciences, Ontario); Dr. Bojana Stefanovic (Sunnybrook Health Sciences, Ontario); Curtis Caldwell, PhD (Sunnybrook Health Sciences, Ontario); Ging Yuek Robin Hsiung, MD, MHSc, FRCPC (U.B.C. Clinic for AD & Related Disorders); Howard Feldman, MD, FRCPC (U.B.C. Clinic for AD & Related Disorders); Benita Mudge, BS (U.B.C. Clinic for AD & Related Disorders); Michele Assaly, MA Past (U.B.C. Clinic for AD & Related Disorders); Andrew Kertesz, MD (Cognitive Neurology St. Joseph’s, Ontario); John Rogers, MD (Cognitive Neurology St. Joseph’s, Ontario); Dick Trost, PhD (Cognitive Neurology St. Joseph’s, Ontario); Charles Bernick, MD (Cleveland Clinic Lou Ruvo Center for Brain Health); Donna Munic, PhD (Cleveland Clinic Lou Ruvo Center for Brain Health); Diana Kerwin, MD (Northwestern University); Marek Marsel Mesulam, MD (Northwestern University); Kristine Lipowski, BA (Northwestern University); Chuang Kuo Wu, MD, PhD (Northwestern University); Nancy Johnson, PhD (Northwestern University); Carl Sadowsky, MD (Premiere Research Inst (Palm Beach Neurology)); Walter Martinez, MD (Premiere Research Inst (Palm Beach Neurology)); Teresa Villena, MD (Premiere Research Inst (Palm Beach Neurology)); Raymond Scott Turner, MD, PhD (Georgetown University Medical Center); Kathleen Johnson, NP (Georgetown University Medical Center); Brigid Reynolds, NP (Georgetown University Medical Center); Reisa A. Sperling, MD (Brigham and Women’s Hospital); Keith A. Johnson, MD (Brigham and Women’s Hospital); Gad Marshall, MD (Brigham and Women’s Hospital); Meghan Frey (Brigham and Women’s Hospital); Jerome Yesavage, MD (Stanford University); Joy L. Taylor, PhD (Stanford University); Barton Lane, MD (Stanford University); Allyson Rosen, PhD (Stanford University); Jared Tinklenberg, MD (Stanford University); Marwan N. Sabbagh, MD (Banner Sun Health Research Institute); Christine M. Belden, PsyD (Banner Sun Health Research Institute); Sandra A. Jacobson, MD (Banner Sun Health Research Institute); Sherye A. Sirrel, MS (Banner Sun Health Research Institute); Neil Kowall, MD (Boston University); Ronald Killiany, PhD (Boston University); Andrew E. Budson, MD (Boston University); Alexander Norbash, MD (Boston University); Patricia Lynn Johnson, BA (Boston University); Thomas O. Obisesan, MD, MPH (Howard University); Saba Wolday, MSc (Howard University); Joanne Allard, PhD (Howard University); Alan Lerner, MD (Case Western Reserve University); Paula Ogrocki, PhD (Case Western Reserve University); Leon Hudson, MPH (Case Western Reserve University); Evan Fletcher, PhD (University of California, Davis Sacramento); Owen Carmichael, PhD (University of California, Davis Sacramento); John Olichney, MD (University of California, Davis Sacramento); Charles DeCarli, MD (University of California, Davis Sacramento); Smita Kittur, MD (Neurological Care of CNY); Michael Borrie, MB ChB (Parkwood Hospital); T Y Lee, PhD (Parkwood Hospital); Dr. Rob Bartha, PhD (Parkwood Hospital); Sterling Johnson, PhD (University of Wisconsin); Sanjay Asthana, MD (University of Wisconsin); Cynthia M. Carlsson, MD (University of Wisconsin); Steven G. Potkin, MD (University of California, Irvine BIC); Adrian Preda, MD (University of California, Irvine BIC); Dana Nguyen, PhD (University of California, Irvine BIC); Pierre Tariot, MD (Banner Alzheimer’s Institute); Adam Fleisher, MD (Banner Alzheimer’s Institute); Stephanie Reeder, BA (Banner Alzheimer’s Institute); Vernice Bates, MD (Dent Neurologic Institute); Horacio Capote, MD (Dent Neurologic Institute); Michelle Rainka, PharmD, CCRP (Dent Neurologic Institute); Douglas W. Scharre, MD (Ohio State University); Maria Kataki, MD, PhD (Ohio State University); Anahita Adeli, MD (Ohio State University); Earl A. Zimmerman, MD (Albany Medical College); Dzintra Celmins, MD (Albany Medical College); Alice D. Brown, FNP (Albany Medical College); Godfrey D. Pearlson, MD (Hartford Hosp., Olin Neuropsychiatry Research Center); Karen Blank, MD (Hartford Hosp., Olin Neuropsychiatry Research Center); Karen Anderson, RN (Hartford Hosp., Olin Neuropsychiatry Research Center); Robert B. Santulli, MD (Dartmouth Hitchcock Medical Center); Tamar J. Kitzmiller (Dartmouth Hitchcock Medical Center); Eben S. Schwartz, PhD (Dartmouth Hitchcock Medical Center); Kaycee M. Sink, MD, MAS (Wake Forest University Health Sciences); Jeff D. Williamson, MD, MHS (Wake Forest University Health Sciences); Pradeep Garg, PhD (Wake Forest University Health Sciences); Franklin Watkins, MD (Wake Forest University Health Sciences); Brian R. Ott, MD (Rhode Island Hospital); Henry Querfurth, MD (Rhode Island Hospital); Geoffrey Tremont, PhD (Rhode Island Hospital); Stephen Salloway, MD, MS (Butler Hospital); Paul Malloy, PhD (Butler Hospital); Stephen Correia, PhD (Butler Hospital); Howard J. Rosen, MD (UC San Francisco); Bruce L. Miller, MD (UC San Francisco); Jacobo Mintzer, MD, MBA (Medical University South Carolina); Kenneth Spicer, MD, PhD (Medical University South Carolina); David Bachman, MD (Medical University South Carolina); Elizabether Finger, MD (St. Joseph’s Health Care); Stephen Pasternak, MD (St. Joseph’s Health Care); Irina Rachinsky, MD (St. Joseph’s Health Care); John Rogers, MD (St. Joseph’s Health Care); Andrew Kertesz, MD (St. Joseph’s Health Care); Dick Drost, MD (St. Joseph’s Health Care); Nunzio Pomara, MD (Nathan Kline Institute); Raymundo Hernando, MD (Nathan Kline Institute); Antero Sarrael, MD (Nathan Kline Institute); Susan K. Schultz, MD (University of Iowa College of Medicine, Iowa City); Laura L. Boles Ponto, PhD (University of Iowa College of Medicine, Iowa City); Hyungsub Shim, MD (University of Iowa College of Medicine, Iowa City); Karen Elizabeth Smith, RN (University of Iowa College of Medicine, Iowa City); Norman Relkin, MD, PhD (Cornell University); Gloria Chaing, MD (Cornell University); Lisa Raudin, PhD (Cornell University); Amanda Smith, MD (University of South Floriday: USF Health Byrd Alzheimer’s Institute); Kristin Fargher, MD (University of South Floriday: USF Health Byrd Alzheimer’s Institute); Balebail Ashok Raj, MD (University of South Floriday: USF Health Byrd Alzheimer’s Institute).
