# Supplementary material for: Neuroinflammation modifies the relationship between stress and perivascular spaces in an elderly population with different levels of cognitive impairment
Source: Front Cell Neurosci. 2024 Nov 13;18:1480405. doi: 10.3389/fncel.2024.1480405 (PMC11603360; doi:10.3389/fncel.2024.1480405)
Supplement: Supplementary file 1 [file Data_Sheet_1.PDF]

Appendix

Section 1: Methods

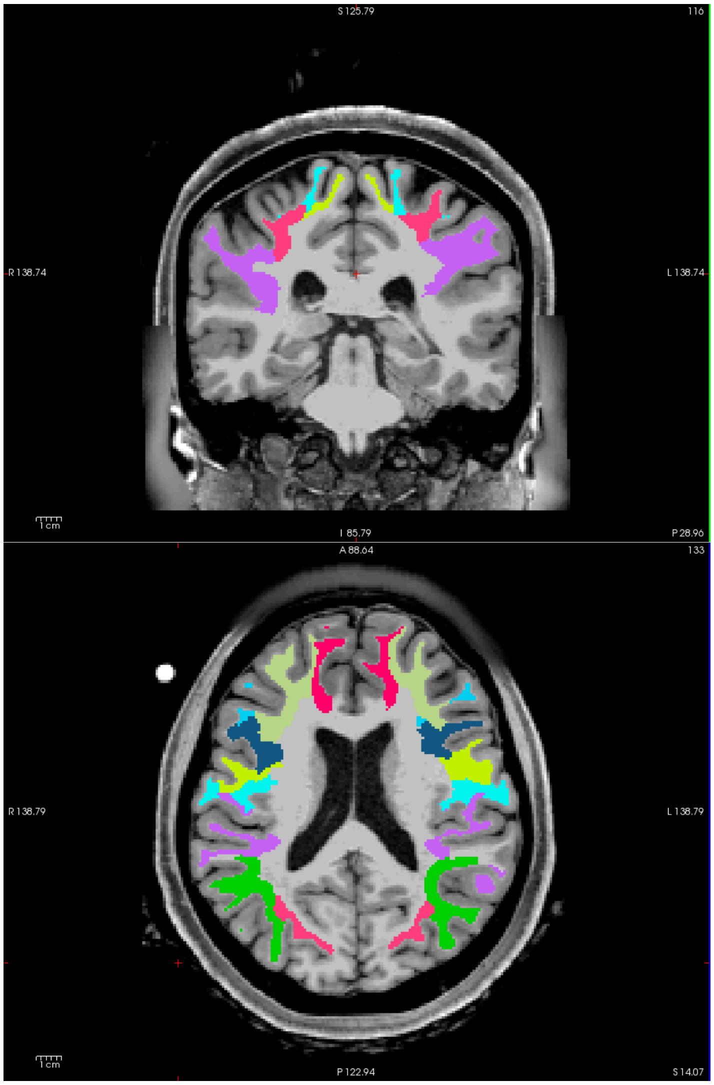

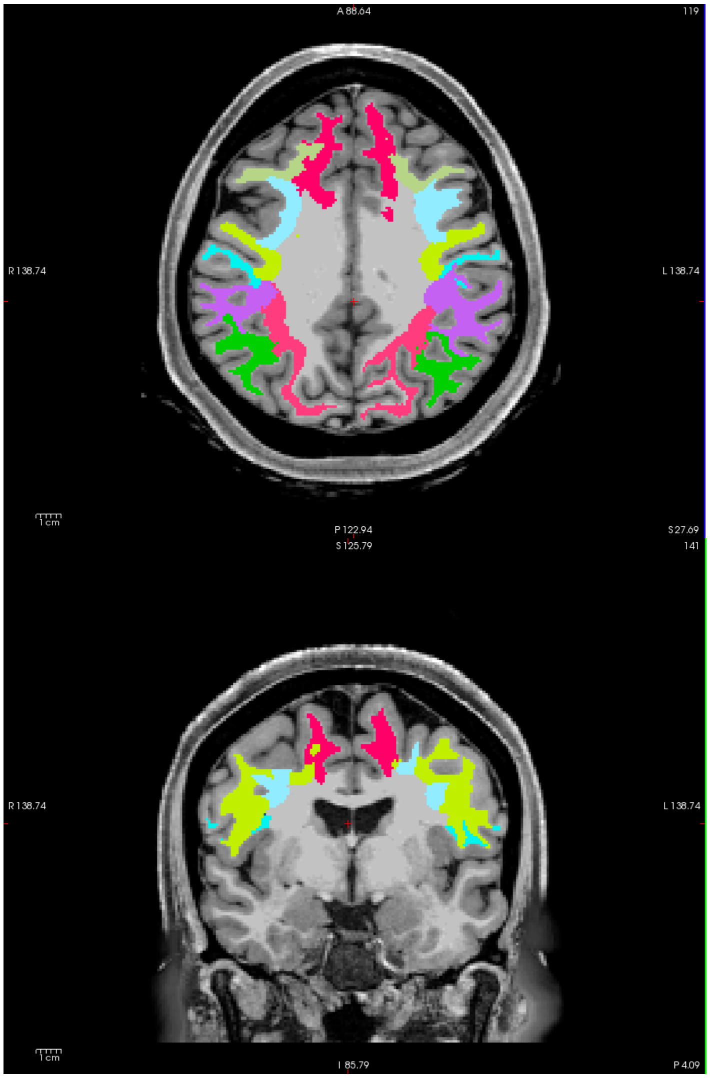

Figure A1: Centrum semiovale mask overlayed on T1w.

**Labels**	**ROI Name**
3003	wm-lh-caudalmiddlefrontal
3008	wm-lh-inferiorparietal

3018	wm-lh-parsopercularis
3019	wm-lh-parsorbitalis
3020	wm-lh-parstriangularis
3022	wm-lh-postcentral
3024	wm-lh-precentral
3027	wm-lh-rostralmiddlefrontal
3028	wm-lh-superiorfrontal
3029	wm-lh-superiorparietal
3031	wm-lh-supramarginal
4003	wm-rh-caudalmiddlefrontal
4008	wm-rh-inferiorparietal
4018	wm-rh-parsopercularis
4019	wm-rh-parsorbitalis
4020	wm-rh-parstriangularis
4022	wm-rh-postcentral

4024	wm-rh-precentral
4027	wm-rh-rostralmiddlefrontal
4028	wm-rh-superiorfrontal
4029	wm-rh-superiorparietal
4031	wm-rh-supramarginal

Table A1: Regions of interest (ROIs) to construct Centrum Semiovale region using FreeSurfer parcellation mask. The white matter labels are based on Desikan-Killiany-Tourville adult cortical parcellation atlas (Klein and Tourville, 2012). Left hemisphere denoted by `wm-lh` has 30XX added to the Label index, and the right hemisphere denoted by `wm-rh` has 40XX.

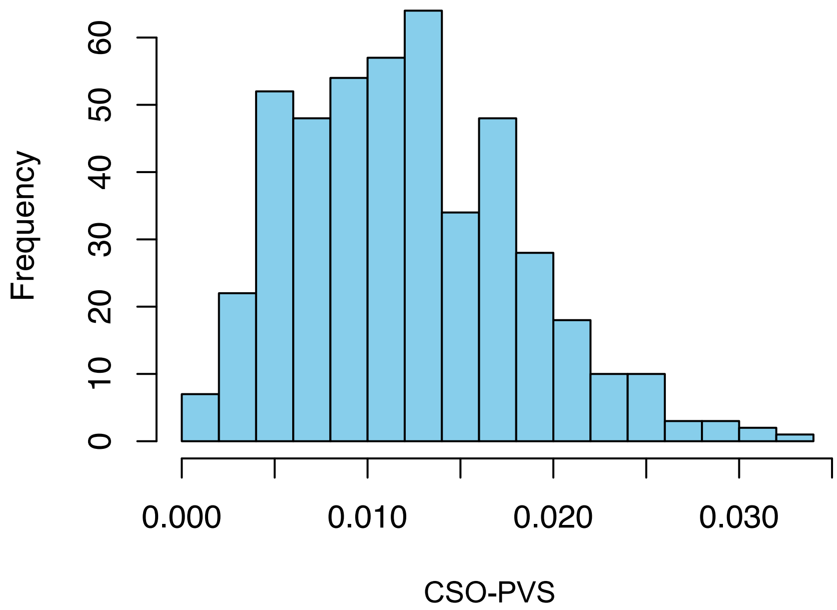

a)

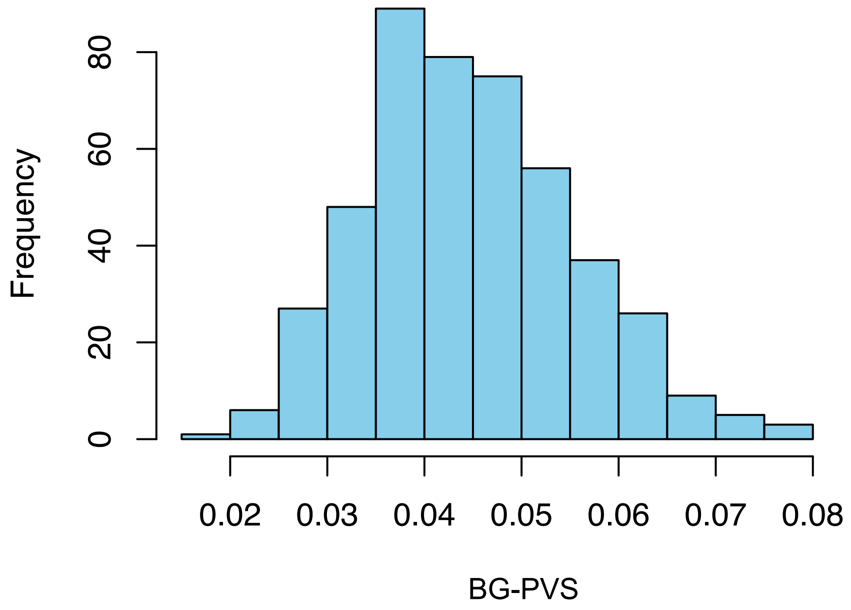

b)

Figure A2: Distribution of the PVS in the centrum semiovale (a) and basal ganglia (b) for the whole sample at 1.5T.

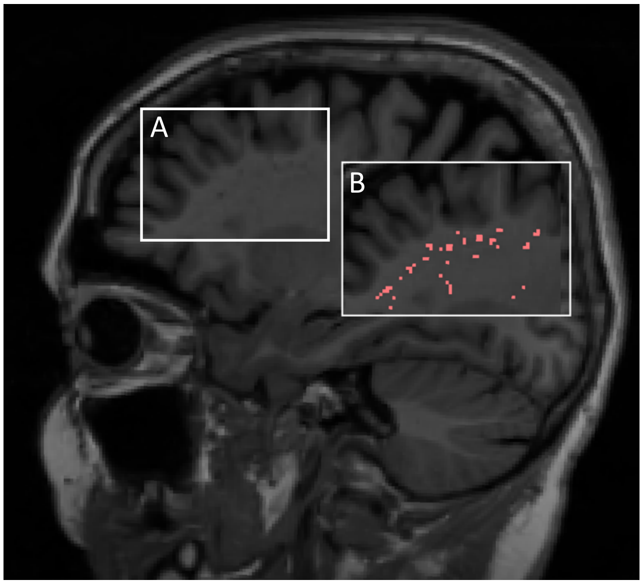

Figure A3: Visual representation of PVS segmentation on a T1-weighted image of an old healthy control participant (sagittal plane). The white box (A) defines the frontal PVS area of interest. The white box (B) shows the PVS mask overlay in pink.

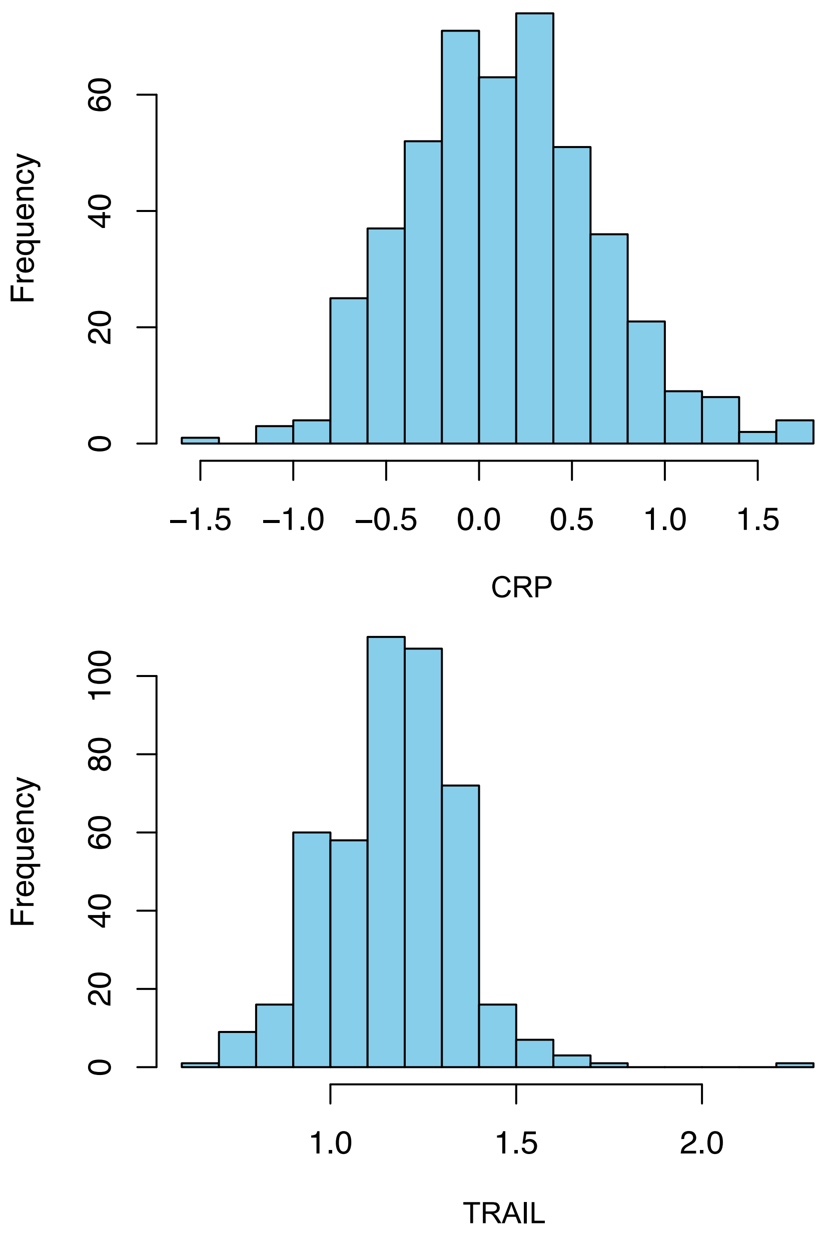
Frequency
0
20
40
60
−1.5
−1.0
−0.5
0.0
0.5
1.0
1.5
CRP
Frequency
0
20
40
60
80
100
1.0
1.5
2.0
TRAIL

a)

b)

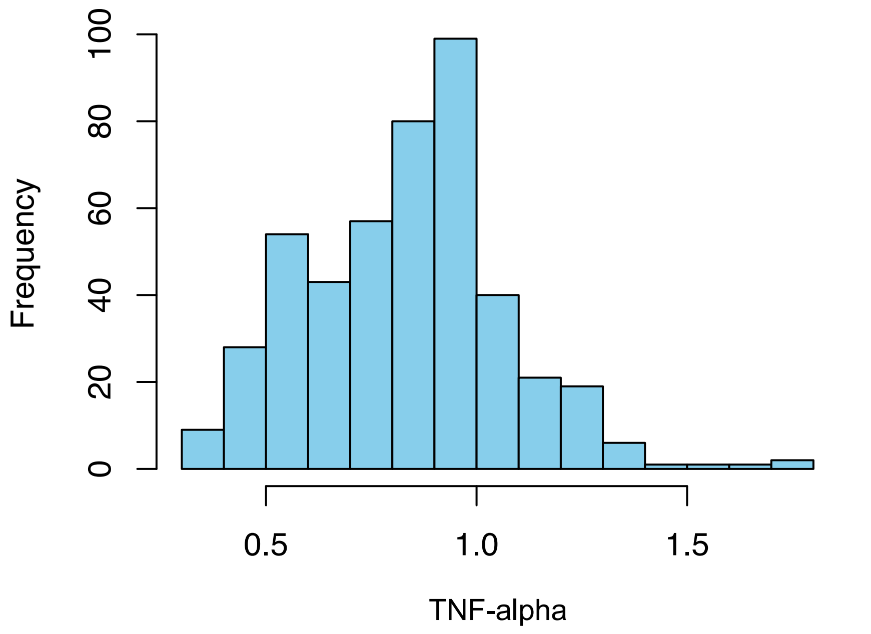

Frequency
0
20
40
60
80
100
0.5
1.0
1.5
TNF-alpha

c)

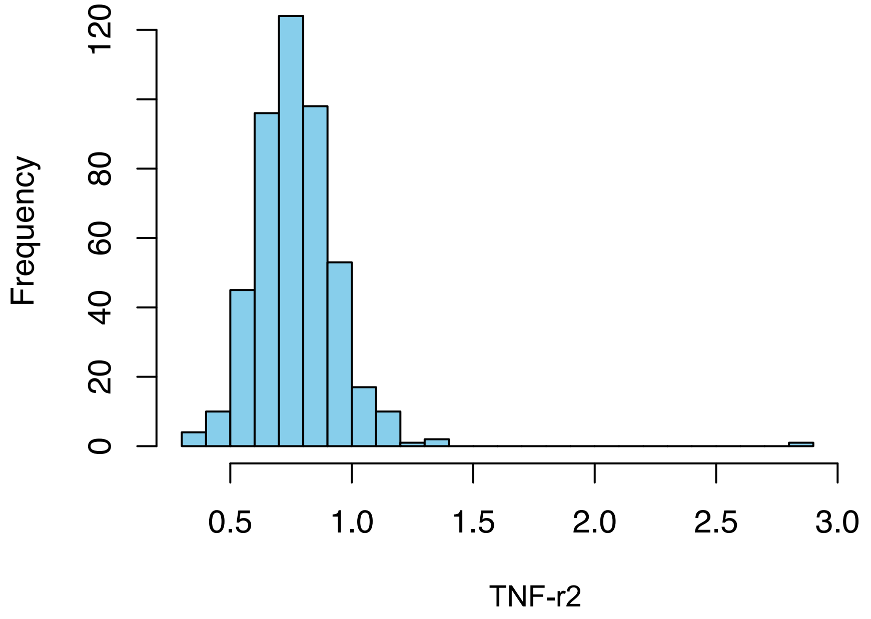

Frequency
0
20
40
60
80
120
0.5
1.0
1.5
2.0
2.5
3.0
TNF-r2

d)

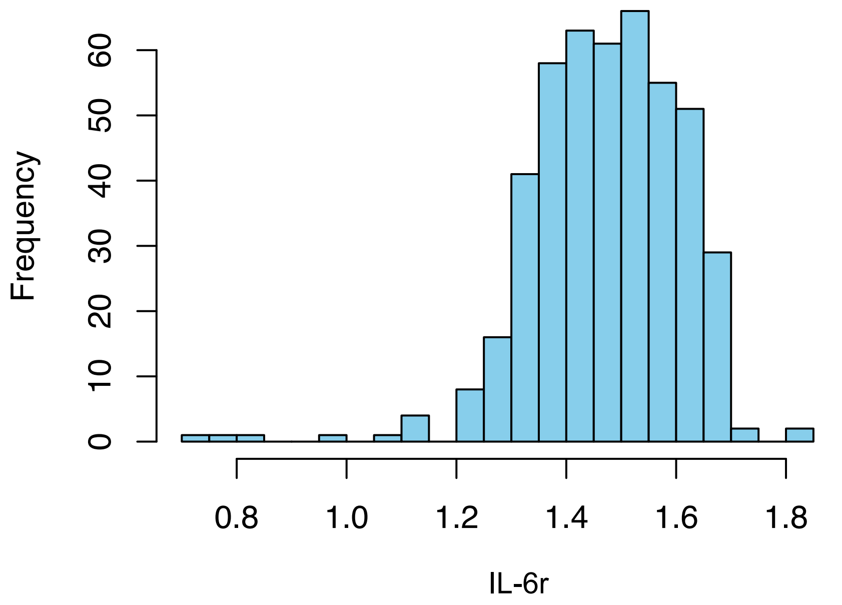
Frequency
0
10
20
30
40
50
60
0.8
1.0
1.2
1.4
1.6
1.8
IL-6r

e)

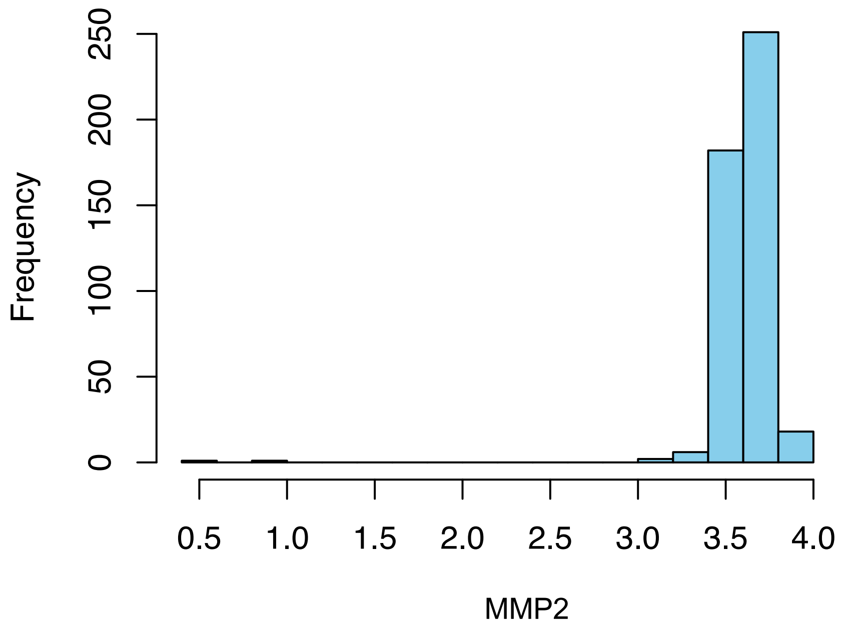
Frequency
0
50
100
150
200
250
0.5
1.0
1.5
2.0
2.5
3.0
3.5
4.0
MMP2

f)

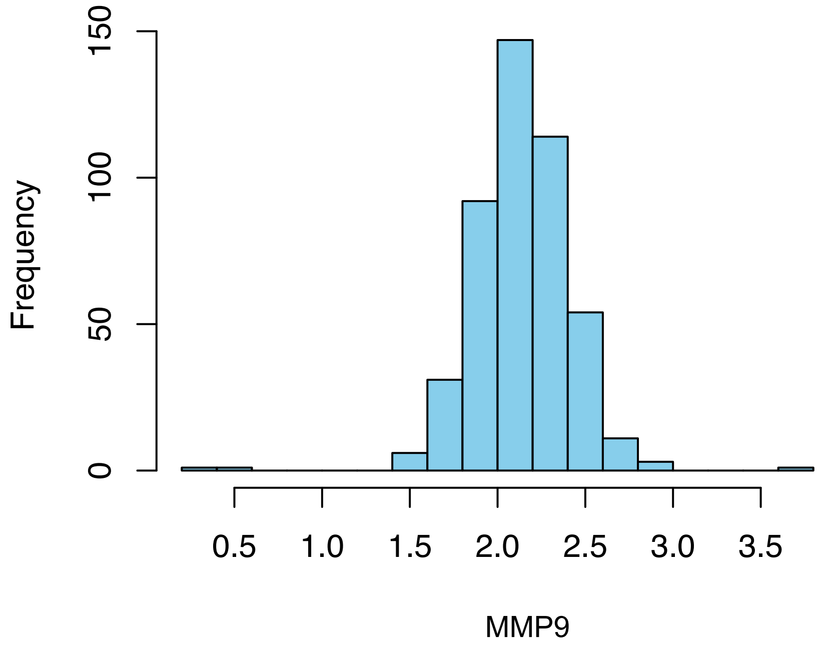

g)

Figure A4: Distribution of inflammatory biomarkers in the 1.5T cohort. a) CRP, b) TRAIL, c) TNF-alpha, d) TNFr2, e) IL6r, f) MMP-2 and g) MMP-9.

Section 2: Results

	Centrum semiovale		
Predictors	*Estimates*	*std. Beta*	*p*
**(Intercept)**	0.00	0.01	**<0.001**
**Cortisol**	1.12	1.01	0.542
**ACE**	1.04	1.01	0.825
**AGE**	1.02	1.13	**<0.001**
**SEX [Male]**	1.02	1.02	0.730
**BMI**	1.01	1.04	0.100
**Total HC**	1.00	1.07	**0.008**

	Basal ganglia		
Predictors	*Estimates*	*std. Beta*	*p*
**(Intercept)**	0.01	0.04	**<0.001**
**Cortisol**	0.91	0.99	0.238
**ACE**	1.13	1.02	0.071
**AGE**	1.01	1.07	**<0.001**
**SEX [Male]**	1.01	1.01	0.755
**BMI**	1.01	1.04	**<0.001**
**Total HC**	1.00	1.01	0.204

Table A2: Negative results of the associations between CSO-PVS and BG-PVS with cortisol

Section 3: **Within-group results**

Demographic tables and results of within-group analyses. Standardized beta coefficients and p-values are reported in CN a), MCI b) and AD c).

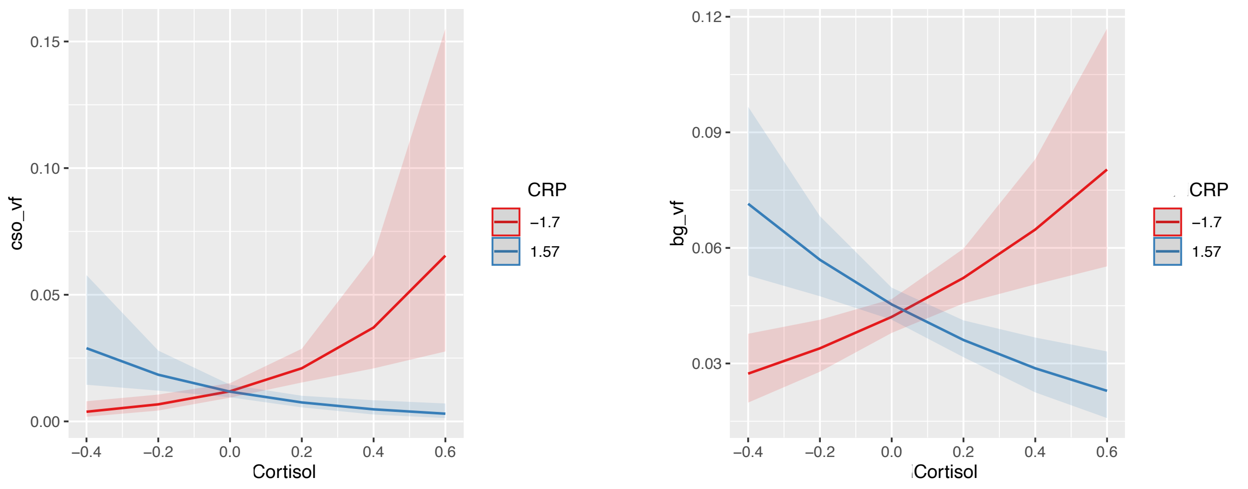

Figure A5: Interaction plots for the MCI group. NOTE: *Plots indicate model-fitted cortisol-PVS curves at two different levels of each inflammatory biomarker. The two levels of inflammatory markers indicate the minimum and the maximum values. Shaded areas represent 95% confidence intervals.
